# Querying clinical data in HL7 RIM based relational model with morph-RDB

**DOI:** 10.1186/s13326-017-0155-8

**Published:** 2017-10-05

**Authors:** Freddy Priyatna, Raul Alonso-Calvo, Sergio Paraiso-Medina, Oscar Corcho

**Affiliations:** 1 Ontology Engineering Group, Universidad Politécnica de Madrid, Madrid, Spain; 2 Biomedical Informatics Group, Universidad Politécnica de Madrid, Madrid, Spain

**Keywords:** Clinical data, R2RML, SPARQL

## Abstract

**Background:**

Semantic interoperability is essential when carrying out post-genomic clinical trials where several institutions collaborate, since researchers and developers need to have an integrated view and access to heterogeneous data sources. One possible approach to accommodate this need is to use RDB2RDF systems that provide RDF datasets as the unified view. These RDF datasets may be materialized and stored in a triple store, or transformed into RDF in real time, as virtual RDF data sources. Our previous efforts involved materialized RDF datasets, hence losing data freshness.

**Results:**

In this paper we present a solution that uses an ontology based on the HL7 v3 Reference Information Model and a set of R2RML mappings that relate this ontology to an underlying relational database implementation, and where morph-RDB is used to expose a virtual, non-materialized SPARQL endpoint over the data.

**Conclusions:**

By applying a set of optimization techniques on the SPARQL-to-SQL query translation algorithm, we can now issue SPARQL queries to the underlying relational data with generally acceptable performance.

## Introduction

In the last years, clinical trials have started introducing genomic variables [1]. This requires performing patient stratification when selecting the patient population to apply the clinical trials to. It involves the use of biomarkers to create subsets within a patient population that provide more detailed information about how the patient will respond to a given drug. Several datasets, commonly produced by different institutions and hence rather heterogeneous in general, need to be used for patient stratification [2]. Interoperability among those datasets is made easier by the use of biomedical standards and terminologies [3]. However, achieving such interoperability poses relevant technological challenges [4]. In this work, we focus on a semantic interoperability approach to homogenize different data models into one Common Data Model (CDM). For this task several projects such as HL7 Reference Information Model (RIM) [5], i2b2 [6], OMOP [7] or CaGRID [8] have defined their own CDM capable of storing heterogeneous data coming from different sources. The basis of the work presented in this paper is founded on the semantic interoperability layer developed in the EURECA project [9], which has been deployed and tested in several healthcare institutions, such as the Institut Jules Bordet [10], the MAASTRO Clinic [11], and the German Breast Group [12].

In previous works [13] we already presented a HL7 RIM [5] relational database implementation used as a CDM in the EURECA semantic interoperability layer. This database aims to facilitate the interconnection with other data sources where medical ontologies are also being used, and has already been used for providing some form of interoperability among real data sources [13] from the aforementioned institutions. We are currently developing ontology-based support to data access to facilitate such integration and allow incorporating other datasets more easily. This is the reason why we were looking into using a Relational Database to RDF (RDB2RDF) solution. We also provide a SPARQL endpoint to a virtual view so that users are relieved from knowing the underlying schema of the implemented database.

RDB2RDF mappings are used to expose data from relational databases as RDF datasets. Two major types of data access mechanisms are normally provided by RDB2RDF tools: i) data translation (a specific case of ETL - Extract, Transform, Load -), where data are materialized into RDF datasets and stored in a triple store (e.g., Virtuoso), which provides a SPARQL endpoint; and ii) query translation, where SPARQL queries are directly translated into SQL according to the specified RDB2RDF mappings, and evaluated against the relational database, and where results are translated back using the mappings to conform with the SPARQL query. In our case, we are interested in using RDB2RDF mappings to make the data stored in our SQL implementation available according to an ontology that reflects the HL7 version 3 RIM. Furthermore, we have a strong requirement to use a query translation approach, given the importance of having fresh results, which cannot always be ensured in the data translation approach.

Our first attempt [14] at applying RDB2RDF-based query translation was with D2R server and mappings [15]. This approach was not applicable since the evaluation of the SQL queries resulting from query translation was not efficient enough. Moreover, in some cases, queries could not be executed by the database management system (e.g., their length was excessive). This was already mentioned in [16] which describes the experience of using RDB2RDF tools in the domain of astronomy. The conclusion there was that RDB2RDF tools were not feasible to be used in such a context, and this conclusion was consistent with our first attempt.

Later, we started using morph-RDB [17] with R2RML mappings [18] for this purpose. We have obtained better results that make this approach applicable in our context. In this paper we describe our experience, which shows that it is possible to use efficient RDB2RDF tools in the medical domain.

This paper is organized as follows. In the “Background” section we discuss our current model for storing medical data, the HL7 RIM ontology, the R2RML mapping language, and our query translation engine morph-RDB. In the “Methods” section we discuss our methodology for mapping legacy data into the HL7 RIM ontology, selection of SPARQL queries for that ontology, and some optimization techniques that have been implemented in morph-RDB. In the “Results and discussion” section, we present our evaluation. Finally in the “Conclusions” section, we provide some conclusions and describe some of our future work in this area, including our deployment plans in the aforementioned healthcare institutions.

## Background

In this section we will review the main foundations of the work that we present in the paper, namely HL7 and the HL7 RIM, the R2RML language, and morph-RDB.

### HL7 RIM

Recent years have witnessed a huge increase of biomedical databases [19]. This increased availability opens up new opportunities, while setting some new important challenges, especially with respects to their integration, which is crucial to obtain a proportional increment of knowledge in the biomedical area. In this context, it is common to establish a CDM for the representation of biomedical data which allow exploiting multiple established terminologies to build a core concept dataset as the common medical vocabulary of the platform

**Fig. 1:**
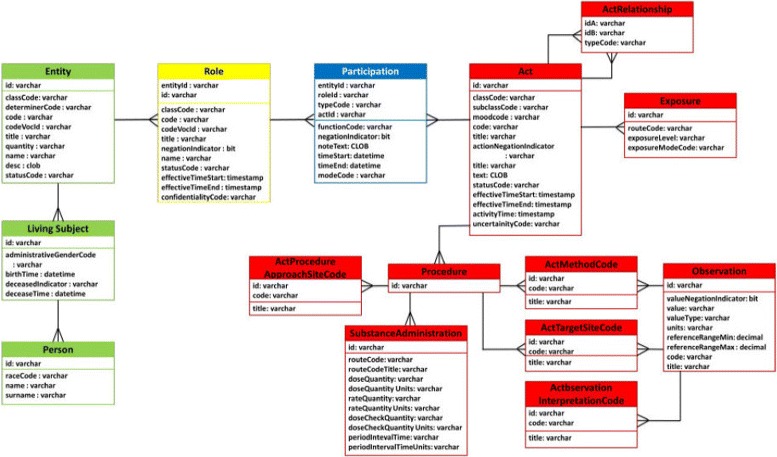
Relational model of H7RIM. Our database schema implementing the HL7RIM model [13]

Among the many Detailed Clinical Models that have been reviewed for the integration of biomedical datasets [20], the HL7 v3 is one of the most relevant, since main requirement for the CDM is that any data coming from clinical institutions can be represented without loss of information. The HL7 RIM offers a wide coverage for representing clinical data and has proven useful for clinical information exchange. The HL7 v3 standard defines the RIM at its core. This definition consists of a UML class diagram (it does not define a data structure or a database model). Besides, issues such as the management of data types are not trivially translatable into a database model. As a consequence, we previously defined a relational model for it, which can be seen in Fig. 1 and described in [13].

The HL7 RIM backbone contains three main classes: Act, Role and Entity, which are linked together by three association classes (Act-Relationship, Participation and RoleLink). The core of the HL7 RIM is the Act class. An Act is defined as “a record of an event that has happened or may happen”. Any healthcare situation and all information concerning it should be describable using the RIM by including the type of act (what happens), the actor who performs the deed and the objects or subjects Entity that the act affects to Role. Some additional information may be provided to indicate location (where), time (when), manner (how), together with reasons (why) or motives (what for). Act and Entity classes have some specializations that add some attributes, such as Observation (a subclass of Act), or Person (a subclass of Entity).

This standard is able to represent almost any healthcare situations and a wide variety of information associated with it [21]. Based on this idea, we have defined a subset of the HL7 RIM schema where we implement the classes and attributes that are necessary to represent the scenario for sharing clinical breast cancer clinical trials data:

- Act, with the subclasses Observation, Procedure, SubstanceAdministration, and Exposure.
- Role.
- Entity, with the sub-classes LivingSubject, Person, and Device.
- The classes; i) ActProcedureApproachSiteCode, ii) ActMethodCode,
  iii) ActTargetSiteCode, iv) ActObservationInterpretationCode, and v) ActObservationValues related to Act.

Attribute data types are rather complex on the RIM, so they are changed according to the mentioned scenario, following HL7 datatype specifications [22]. Therefore some attributes were simplified in the relational model compared to those defined by HL7 v3 standard. To improve performance and understanding of the HL7 RIM schema, it is defined a set of views. These views cover the access retrieval requirements for the clinical scenario. We defined a view for each clinical contexts (Observation, Procedure, SubstanceAdministration, and Exposure).

Therefore, the defined HL7 RIM-based CDM above fulfills the requirements needed for breast cancer clinical trials scenario. Furthermore, we have created an ontology that reflects the HL7RIM model [23], which is available for others to reuse.

**Fig. 2:**
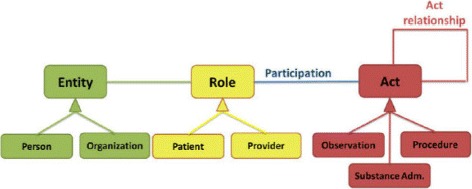
Simplified HL7RIM model. Our simplified logical database schema implementing the HL7RIM model

**Fig. 3:**
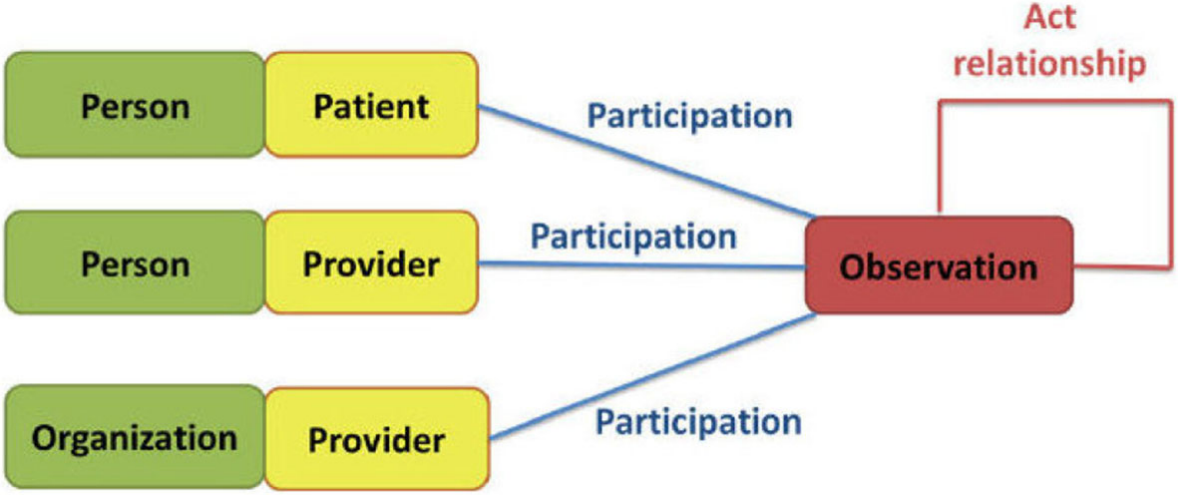
Logical view of HL7RIM model. Logical view of observation data in the HL7RIM model

Figure 2 depicts a simplified schema of the implemented database following the HL7 v3 RIM definition. However, typically relationships among Entity and Role instances are one-to-one. Moreover, the Act table is the backbone but data is classified as one of its descendants (Observation, Procedure, Substance Administration, Exposure, etc.). Thus the logical schema for querying an Act descendant (i.e. Observation) from our database looks like the schema represented in Fig. 3.

Therefore, every Act subclass in the HL7 v3 RIM data schema can be represented as a star diagram — typically used in data warehouse definition. Our database can be visualized as a snowflake diagram similar to the i2b2 star model [6]. Each event record will be a subclass of Act (similarly to the i2b2 fact table). Entities and Roles (patient, location, care provider, etc.) are lookup tables called Dimensions.

Conversely to other works in literature that use query translation [8], since Act tables contain the biggest amount of data in the model, we have adopted the approach of dividing complex queries into atomic queries. Consequently, in order to efficiently execute queries involving several instances of acts and relationships (e.g. temporal dependencies), these queries are divided and results are later combined using set operators [13].

### R2RML

R2RML [18] is a W3C recommendation for the definition of a mapping language from relational databases to RDF. An R2RML mapping document consists of a set of Triples Maps rr:TriplesMap, used to specify the rules to generate RDF triples from database rows/values. A TriplesMap consists of:

- A logical table rr:LogicalTable that is either a base table or SQL view, used to provide the rows to be mapped as RDF triples.
- A subject map rr:SubjectMap that is used to specify the rules to generate the subject component of RDF triples.
- A set of predicate object maps rr:PredicateObjectMap that is composed by a set of predicate maps rr:PredicateMap and object maps rr:ObjectMap (to generate the predicate and object components of RDF triples, respectively). If a join with another triples map is needed, a reference object map rr:RefObjectMap can be used. The other triples map to be joined is specified in rr:parentTriplesMap and the join condition is specified via rr:Join

**Fig. 4:**
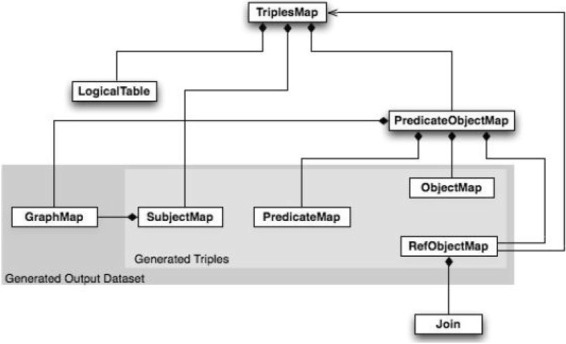
R2RML TriplesMap overview. An overview of R2RML TriplesMap, taken from [18]

Figure 4 illustrates an overview of an R2RML TriplesMap class.

**Fig. 5:**
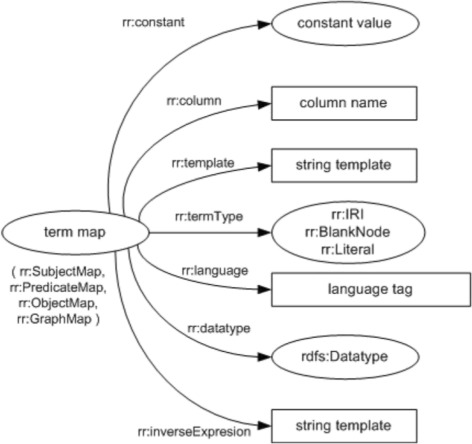
R2RML TermMap overview. An overview of R2RML TermMap, taken from [18]

Subject maps, predicate maps, and object maps are term maps, which are used to specify rules to generate the corresponding RDF triples element, and those rules can be specified as a constant rr:constant, a database column rr:column, or a template rr:template. Figure 5 illustrates an overview of an R2RML TermMap class.

### morph-RDB

morph-RDB is part of the morph suite [24]. It receives as an input the connection details to a relational database, an R2RML mapping document and a SPARQL query. It translates the SPARQL query into the underlying relational database and translates the results back into a format appropriate for the SPARQL query. The query translator component in morph-RDB implements the algorithm described in [17], which extends previous work in [25] that defined a set of mappings and functions in order to translate SPARQL queries posed against RDB-backed triples stores into SQL queries, prove the correctness of the query translation using the notion semantic-preserving. In other words, the SPARQL query realized as an SQL query returns the same answers as the same SPARQL query executed over an R2RML materialization. We extend their work by relating those mappings and functions with the R2RML mapping elements.

For an in-depth explanation of the query rewriting algorithm, we recommend the aforementioned references. As a quick summary, we use the following mappings and functions:

- *α* mapping, which given a triple pattern *tp* and an R2RML mapping document *m*, returns the corresponding logical tables associated to the pattern.
- *β* mapping, which given a triple pattern *tp* and an R2RML mapping document *m*, returns the corresponding columns associated to the component of the triple pattern (subject, predicate, or object).
- *name* function, which generates a unique alias for the projected attributes.
- *genPRSQL* function, which given a triple pattern *tp*, the *β* and *name* functions, and an R2RML mapping document *m*, generates a SQL expression that projects only the attributes returned by the *beta* mapping and renames them using the *name* function.
- *genCondSQL* function, which given a triple pattern *tp* and an R2RML mapping document *m*, generates an SQL expression (returning only non-null values for that columns returned by *β* mapping using “IS NOT NULL” expression) that filters the logical tables returned by *α* to match the triple pattern *tp*.
- *trans* function, which given a SPARQL graph pattern (triple pattern, AND, OPT, UNION, FILTER, SELECT) and an R2RML mapping document *m*, generates the SQL query that when evaluated, generates the result of the corresponding SPARQL pattern.

The details of the definitions and algorithms defined for the above mappings and functions are provided in [17].

#### **Example 1**

**Fig. 6:**
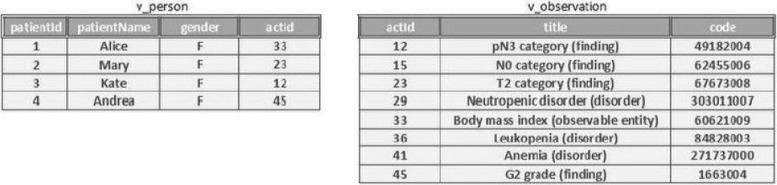
Tables Person and Observation

Consider the following table v_person(patientId, patientName, gender, actId) which stores the information about patients. This table is mapped to the class Patient with the attribute patientId as the identifier (together with base URI for class Patient) of the instances. Attributes patientId and patientName are mapped to ontology properties hasID and hasName, respectively. Now let’s add another table v_observation(actId, title, code) that describes observations. This table mapped to class Observation with actId as the identifier of the instances, and the attribute title mapped to property hasTitle. patientId and actId are primary keys of the tables v_person and v_observation, respectively. Furthermore, the actId of table v_person is a foreign key that refers to the column actId of table v_observation, and this relation is mapped to property hasObservation. The instances of the tables can be seen in Fig. 6.

Consider the following triple pattern tp = (?p :hasPatientName ?pName).

- *α*(tp) = v_person.
- *β*(tp.subject) = v_person.patientId, *β*(tp.predicate) = ’:hasPatientName’, *β*(tp.object) = v_person.patientName.
- *name*(?p) = var_p, *name*(:hasPatientName) = iri_hasPatientName, *name*(*pName*) = var_pName.
- *genPRSQL*(tp) = v_person.patientId AS var_p, ’:hasPatientName’ AS iri_hasPatientName, v_person.patientName AS var_pName
- *genCondSQL*(tp) = v_person.patientId IS NOT NULL AND v_person.patientName IS NOT NULL

Finally, the results returned by the SQL queries obtained as a result of the previous step are the values stored in database servers, and not the RDF terms ones expected as a result of the evaluation of a SPARQL query. This is necessary in order for database servers to be able to exploit indexes over the database values that haven’t been transformed into other values. For example, the result for subject values may come from the primary key columns. Thus, upon receiving the database results that correspond to R2RML template mappings, morph-RDB translates the results according to those mappings.

## Methods

### R2RML mappings creation

We have created an ontology that reflects the HL7 RIM [23], and which has been made available for other teams to reuse. After that, we have started creating the corresponding R2RML mappings, as follows:

1. Mappings to tables. As we use a consistent naming convention when implementing the HL7 RIM in both the ontology and the database schema, we can easily create an initial version of our R2RML mappings using a direct mapping [26] fashion, which is useful for bootstraping the mapping generation task. In this way, mappings between database tables are created for the corresponding class URI, one Triples Map for each table. For example, in Triples Map *TriplesMapAct*, table *act* will be mapped to class *hl7rim:act*, or column *moodCode* which will be mapped to property *hl7rim:act_moodCode* using rr:column. We also mapped joins as instances of RefObjectMap, such as the property *hl7rim:act_procedure* that joins *TriplesMapAct* and *TriplesMapProcedure*. This is done for all the tables except for those having the corresponding views, such as table *person*, which has view *v_person*.
2. We repeat the previous step for the views.
3. Template mappings. Afterwards, we created mappings for those properties whose values cannot be obtained from a single database column. For example, the property hl7rim:observation_refRange, which is mapped using rr:template with *referenceRangeMin - referenceRangeMax* as its template value.

In total, the R2RML mapping document that we use here consists of 20 Triple Maps (6 of them mapped to views instead of tables) and 364 Predicate Object Maps (56 are rr:RefObjectMap that join Triple Maps).

### SPARQL queries collection and grouping

We have collected a total of 45 SPARQL queries that are used in the patient recruitment and cohort selection scenario for breast cancer clinical trials. The complete list of queries and their natural language descriptions are available at https://doi.org/10.6084/m9.figshare.5459572.v1 ^1^. From this query list, we asked our domain experts to group the queries into a five groups and select representative of each group, as shown in Table 1.

**Table 1:** Grouping of all queries according to the representative queries

Representative query	Similar queries	TP	No. of unique subjects	OPT	FILTER
Q01	1, 2, 5, 15, 19, 37, 41, 42	4	2	1	0
Q10	3, 10, 11, 40	25	9	21	4 (IN)
Q14	4, 6, 9, 14, 16, 17, 18, 19, 21, 35, 38, 39, 43	15	6	5	2 (IN, arithmetic)
Q34	22, 23, 24, 25, 26, 27, 28, 29, 30, 31, 32, 33, 34, 44	6	3	1	1 (IN)
Q45	7, 8, 12, 13, 20, 36, 45	14	5	4	2 (IN)

The query characteristics of each representative query are as follows:

- **Demographics query (Q01)**. This query retrieves demographic information about all patients. It contains 4 triple patterns, 2 unique subjects, 1 triple pattern that is inside an OPTIONAL block, and 1 FILTER pattern.
- **Substance administration query (Q10)**. This query retrieves the information of patients who were administered diphosphonate, including the information associated with the target site, the method used, and the approach site, if it exists. It consists of 35 triples patterns with 9 unique subjects. Most of the triple patterns are inside nested OPTIONAL blocks. There are 21 OPTIONAL blocks in this query, some of which are nested under another OPTIONAL block. A FILTER pattern is used to filter results based on a certain condition.
- **Laboratory results query (Q14)**. This query retrieves the information of patients who suffer anemia and whose body mass index is less than or equal to 30. It contains 15 triple patterns, with six unique subjects and 5 OPTIONAL patterns. Furthermore, some of the OPTIONAL blocks are nested inside a parent OPTIONAL block. This query also contains two FILTER patterns to filter results for particular code values and to perform some arithmetic calculations.
- **Procedure query (Q34)**. This query retrieves the information of all patients who were administered chemotherapy. It consists of 6 triple patterns with one of them located inside an OPTIONAL pattern, and one FILTER pattern. There are 3 unique subjects in this query.
- **Observation query (Q45)**. This query retrieves the information of patients who have been detected a category T2 breast tumor. It consists of 14 triple patterns and 5 unique subjects. There are 4 OPTIONAL patterns, one of them nested, and 2 FILTER patterns.

### Query translation optimization

The query translation technique presented above does not necessarily generate optimal SQL queries. Based on the set of SPARQL queries that we have evaluated, we have observed that several patterns occur frequently. Hence we describe optimization techniques that can be applied to these commonly occurring patterns in order to generate more efficient queries.

#### Optimization 1: self-join elimination

A set of triple patterns connected by the AND operator and sharing the same subject occur frequently. We call this pattern *Subject Triple Group* (STG). A common pattern used in SPARQL queries is a set of triple patterns having the same subject,

##### **Definition 1**

A Subject Triple Group (STG) pattern is defined recursively as follows:

- If *tp*_1_ and *tp*_2_ are triple patterns and *tp*_1_.*subject*=*tp*_2_.*subject*, then (*tp*_1_AND*tp*_2_) is an STG pattern.
- If *P*_1_ is an STG pattern, *TP* is a triple pattern, and *P*_1_.*subject*=*TP*.*subject*, then (*P*_1_AND*TP*) is an STG pattern.

Using the *trans* algorithm defined in [17], the process of translating STG patterns having *n* triple patterns is defined by recursively calling the function *trans*(*AND*) of *n*−1 triple patterns with the *n*th triple pattern.

##### **Definition 2**

An STG pattern is translated using the function *trans* recursively as:

- *trans*(*tp*_1_AND*tp*_2_)=*trans*(*tp*_1_)⋈*trans*(*tp*_2_).
- *trans*({*tp*_1_AND*tp*_2_AND⋯AND*tp*_*n*−1_}AND*tp*_*n*_)=*trans*({*tp*_1_AND*tp*_2_AND⋯AND*tp*_*n*−1_})⋈*trans*(*tp*_*n*_).

##### **Example 2**

Consider the following graph pattern *gp*={*tp*1AND*tp*2} where tp1 = (?p :hasPatientID ?pID) and tp2 = (?p :hasPatientName ?pName). The result of translating *gp*, denoted as *trans*(*gp*), is:



where trans(tp1) = (SELECT patientId AS var_p, patientId AS var_pID FROM v_person WHERE patientId IS NOT NULL) and trans(tp2) = (SELECT patientId AS var_p, patientName AS var_pName FROM v_person WHERE patientId IS NOT NULL AND patientName IS NOT NULL).

Records of tables in an RDB2RDF context are already arranged in a tabular fashion. Exploiting this fact, we create *trans* ^*stg*^, a more optimised version of the algorithm *trans* that takes STG patterns and translates them without generating self-joins.

##### **Example 3**

Consider again the SPARQL query in Example 1. With self-join elimination, the result of translating that query, denoted as *trans* ^*stg*^(*gp*), is:



#### Optimization 2: replacing left-outer join with inner join

Another pattern is a *Subject Triple Group with Optional* (OSTG), that is an OPTIONAL pattern that consists only of one triple pattern, preceded by an STG pattern or a triple pattern.

##### **Definition 3**

A Subject Triple Group with Optional (OSTG) is defined recursively as follows:

- If *tp*_1_ and *tp*_2_ are triple patterns, *tp*_1_.*subject*=*tp*_2_.*subject*, and *tp*_2_.*object*∉*var*(*tp*_1_) then (*tp*_1_OPT*tp*_2_) is a Subject Triple Group with Optional where *var*(*P*) refers to a set of variables in the pattern *P* and *P*.*subject* refers to the subject of the pattern *P*.
- If *stg* is a Subject Triple Group, *tp* is a triple pattern, *stg*.*subject*=*tp*.*subject*, and *tp*.*object*∉*var*(*stg*), then (*stg*OPT*tp*) is a Subject Triple Group with Optional.
- If *ostg* is a Subject Triple Group with Optional, *tp* is a triple pattern, *ostg*.*subject*=*tp*.*subject*, and *tp*.*object*∉*var*(*ostg*), then (*ostg*OPT*tp*) is a Subject Triple Group with Optional.

Because the OPTIONAL keyword corresponds to a left outer join, naïvely translating this pattern produces one left-outer join for each OPTIONAL pattern. We extend our query translation technique, so that the optimized query translation generates an inner join, which is cheaper to evaluate than left-outer join, by removing the conditional expression IS NOT NULL corresponding to the function *genCondSQL* of the triple pattern in the OPTIONAL pattern.

##### **Example 4**

Consider the following pattern:



Without any optimizations applied, the result of translating this query is:



By changing the type of join from left-outer to inner, removing the conditional expression name IS NOT NULL, and applying the self-join elimination (O1), the optimized query generated becomes:



#### Optimization 3: phantom triple pattern introduction

##### **Example 5**

Consider the following pattern, which is neither an STG pattern nor an OSTG, thus, none of the aforementioned optimizations can be applied.



In order to exploit the optimisations we have presented so far, this query has to be transformed into another query whose resulting query translation can be optimised. To do that, we use the fact that for every IRI *x*, the fact (*x a rdf:Resource*) holds, so that we can safely add this triple pattern to the query without changing its semantics. We call such triple pattern a *phantom triple pattern*. The result of adding the phantom triple pattern is:



Now with the new pattern that emerged, the optimization for OSTG pattern can be applied.

#### Optimization 4: other optimizations

We also apply additional optimizations such as:

- IS NOT NULL checking. Recall that the function *genCondSQL* generates an IS NOT NULL expression for those columns returned by the *β* mapping, however, the database metadata may contain constraints that certain columns cannot have NULL values, such as primary key columns. Thus, upon reading this constraint, we can safely drop the IS NOT NULL condition in the genCondSQL expression.
- Tables reordering. When the translation query contains multiple joins, we can also read the metadata for the size/number of rows of the joined tables. We reorder the tables so that the smaller tables are joined first. In doing so, we may help the database optimiser to reduce the number of rows to be joins in the intermediate results.
- Union reduction. When an unbounded predicate triple pattern (that is, when a triple pattern has a variable in its predicate component) is translated, all the possible mapped predicates are translated and put together as a UNION query. Depending on the number of properties mapped in the R2RML mapping document, the generated UNION query may be unnecessarily large. In order to reduce the number of elements in the UNION part of the query, it is possible to analyze the combination of mappings and queries whose resulting translation can be safely removed.

##### **Example 6**

Consider the triple pattern tp = (:Patient/1 ?p "Bob"). If the R2RML mapping document contains mappings of predicate :hasPredicateName whose range is a literal, and another predicate :hasObservation whose range is an IRI, then triple pattern will be translated as *trans* (:Patient/1 :hasPatientName "Bob") UNION *trans* (:Patient/1 :hasObservation "Bob"), then we can drop the result of translating (:Patient/1 :hasObservation "Bob") because the range of the predicate :hasObservation does not agree with the literal "Bob".

## Results and discussion

We were interested in comparing morph-RDB with another well-established RDB2RDF engine, such as D2R, considering the total time required for the execution of the SPARQL queries.

The machine used in our evaluation has the following specifications: CPU Intel(R) Xeon(R) CPU E5-2650 0 @ 2.00 GHz, 8 GB of RAM, 750 GB HDD with Ubuntu Server 12.04 and MySQL Server 5.5. The dataset contains information of 3 months of historical clinical data, with 4674 patients and 65056 acts, among many other tables. The total size of the database is 105 MB. This database will be growing in the future, as more data is added as a result of the data integration processes carried out in the context of the projects where the database is being generated.

**Fig. 7:**
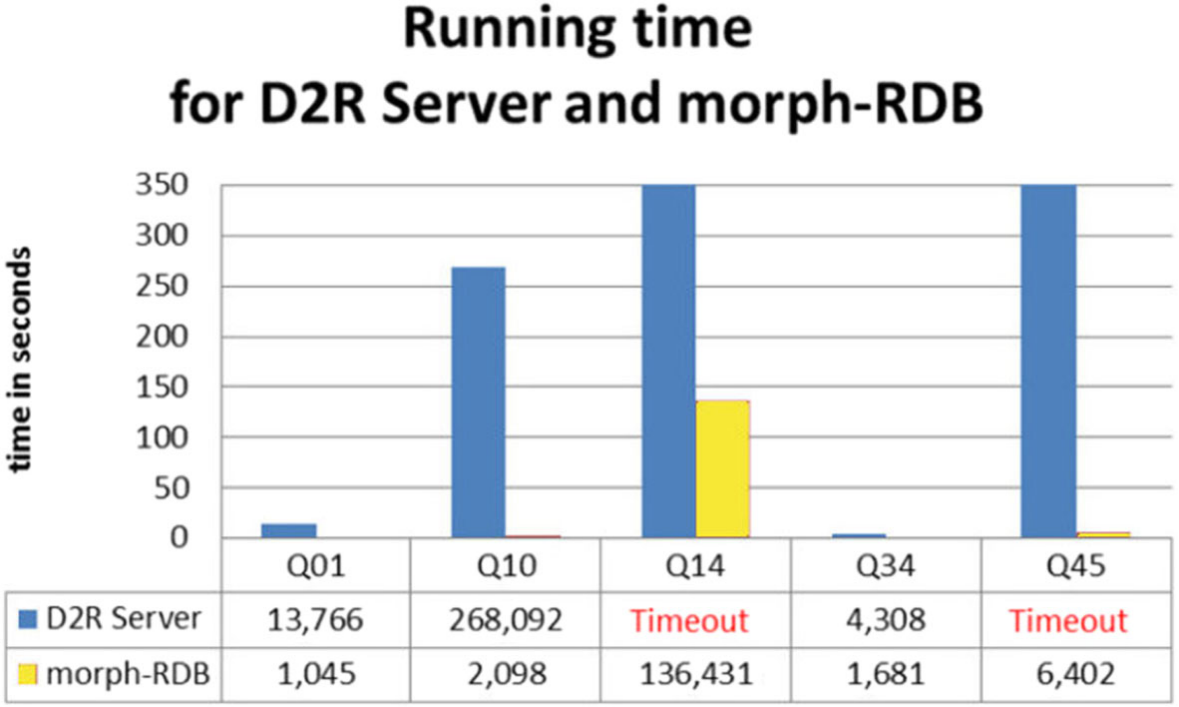
Evaluation queries running time. Running time for our selected queries on morph-RDB and D2R (in seconds)

We include in our calculation the time required to initialize the engine, the time needed for SPARQL-to-SQL query translation, the time needed to evaluate the SQL queries, and the time needed to translate back the result from the database using the mappings into the result expected by the SPARQL queries. Figure 7 provides details for the five selected queries, which are also similar to the results obtained for the other queries in our query set. We can easily see that in most cases our total execution time is much lower than the one required for D2R Server. In some cases (queries Q14 and Q45) D2R Server was not able to produce results in less than five minutes. The results for the rest of the queries are available at the previous link.

We were also interested in how the SQL queries that result from the query rewriting approach perform in comparison to the SQL queries that would have been natively created by a SQL expert. For this reason, we asked a domain expert with good knowledge of the HL7 RIM relational database to construct SQL queries that were semantically equivalent to the corresponding SPARQL queries. In other words, without taking into account the mapping elements, such as template or URI generation, the SPARQL and SQL queries should return the same answer.

We evaluated each query 5 times in cold and warm modes. In the cold mode, we restart the server and empty the cache before we evaluate the next query. In the warm mode, we skip these steps and execute the queries directly one after the another. We measure the averages of query execution time and normalize the query evaluation time to the native query evaluation time. As an additional note, we can only do this type of evaluation using morph-RDB and native queries, as D2R Server produces multiple SQL queries in many cases and performs joins in memory, which makes it not comparable with the native or morph-RDB queries.

**Fig. 8:**
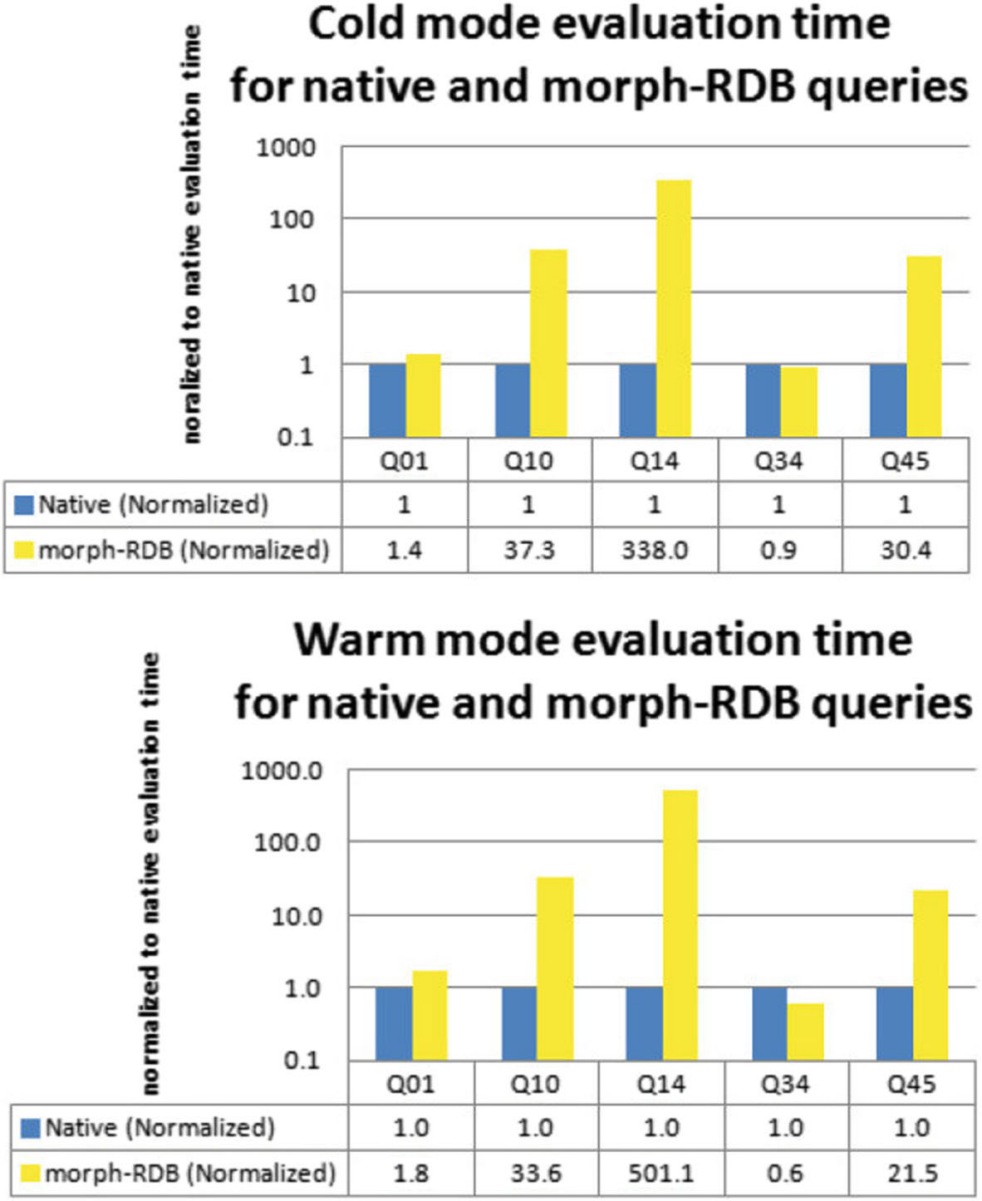
Warm and cold evaluation. Query evaluation time in warm and cold modes

The results from both evaluation modes can be seen in Fig. 8 and they show a similar trend. Furthermore, we observed that in the warm mode, the database server does not lose its capability of reusing previous results of the query cache. This is reflected by the fact that only the first run of the query takes more time to complete, while subsequent queries can be evaluated with only a fraction of that time. Some of those queries produced by morph-RDB can be evaluated in a reasonable time. For example, the resulting query translation of query Q01 can be evaluated in a similar time as the native query Q01. Furthermore, the resulting query translation Q34 can be evaluated in less time than its corresponding native queries, which can be an indicator that there might be still room for improving the corresponding native query. Some other queries, such as Q10 and Q45, need more time to be evaluated, being in the range of 20-35x slower than the corresponding native queries, which we still consider acceptable. The query Q14, however, needs more investigation, as it takes a lot of time to be evaluated, 380-500x slower than the native query. We suspect this is caused by the arithmetic operation that is performed over the resulting translation queries.

## Conclusions

In this paper we have shown that SPARQL queries can be used as a means to query relational clinical data that is integrated into an HL7 version 3 RIM database implementation. We collected a set of 45 real SPARQL queries required by our application domain and that will be deployed in a set of medical institutes, chose five of them as the most representatives ones, and evaluated those queries using D2R Server and morph-RDB as our RDB2RDF tools. We have shown that, in general, we got better results with morph-RDB than D2R Server for accessing relational data using SPARQL.

However, there are still some important remaining challenges to be considered. We still have queries that require too much time to be evaluated, (e.g. query Q14), because of the arithmetic operation that is included in the SPARQL query and in its resulting translation into SQL. Investigating and designing optimizations for dealing with this type of query will be part of our future work.

## Endnote

^1^ Last accessed: May 12th, 2016. If the paper is accepted, they will be uploaded to figshare or zenodo and the link will be changed.

## Abbreviations

CDM: Common data model
ETL: Extract transform load
OSTG: Subject triple group with optional
RDB2RDF: Relational database to RDF
RIM: Reference information model
STG: Subject triple group
